# A survey of barriers and facilitators to ultrasound use in low- and middle-income countries

**DOI:** 10.1038/s41598-023-30454-w

**Published:** 2023-02-27

**Authors:** Amy Sarah Ginsburg, Zylee Liddy, Parsa T. Khazaneh, Susanne May, Farhan Pervaiz

**Affiliations:** 1 Clinical Trials Center, Department of Biostatistics, University of Washington, Building 29, Suite 250, 6200 NE 74th Street, Seattle, WA 98115 USA; 2 Department of Genetic Epidemiology, University of Washington, Seattle, WA USA; 3 Michael G DeGroote School of Medicine, McMaster University, Hamilton, ON Canada; 4 Division of Emergency Medicine, Department of Family Medicine, McMaster University, Hamilton, ON Canada

**Keywords:** Health care, Medical research

## Abstract

Point-of-care ultrasound has the potential to help inform assessment, diagnosis, and management of illness in low- and middle-income countries (LMIC). To better understand current ultrasound use, barriers and facilitators to use, and perceptions and practices in LMIC, we conducted an anonymous online global survey targeting healthcare providers training and using ultrasound in LMIC. A total of 241 respondents representing 62 countries participated and most were physicians working in publicly-funded urban tertiary hospitals in LMIC. Most had received ultrasound training (78%), reported expertise (65%) and confidence (90%) in ultrasound use, and had access to ultrasound (88%), utilizing ultrasound most commonly for procedures and for evaluations of lungs, heart, and trauma. Access to an ultrasound machine was reported as both the top barrier (17%) and top facilitator (53%); other common barriers included access to education and training, cost, and competition for use and other common facilitators included access to a probe, gel, and electricity, and acceptance by healthcare providers, administrators, and patients. Most (80%) noted ultrasound access was important and 96% agreed that ultrasound improves quality of care and patient outcomes. Improving access to low-cost ultrasound equipment is critical to increasing ultrasound use among those who are trained.

## Introduction

Ultrasound has experienced rapid growth and expansion in diagnostic and management applications, evolving from a tool reserved primarily for radiologists and cardiologists into a point-of-care, multi-use technology utilized by both specialists and primary healthcare providers for a myriad of indications in many different practice settings^1,2^. A dynamic tool allowing imaging in real-time without risk of ionizing radiation exposure, ultrasound is used frequently throughout emergency, outpatient, and inpatient settings in high-income countries (HIC) and has become a mainstay in modern clinical practice^1,2^. As ultrasound has become less expensive and more durable and portable, availability and access to ultrasound has increased globally, including in some low- and middle-income countries (LMIC), where use cases have included echocardiography, obstetrics, lung, and trauma among many others^3–12^. Requiring less infrastructure, human resources, time, and expense than other imaging modalities, the introduction of ultrasound to LMIC has the potential to improve patient diagnosis and management and resource utilization^2,6,11,13^. In mobile clinics in rural Uganda, imaging with ultrasound facilitated confirmation of a suspected initial diagnosis in 50% of cases and supported a change in initial diagnosis in 23% of cases^14^. In hospitals in Rwanda, point-of-care ultrasound use changed medications administered in 42% of cases and admission decisions in 30% of cases^15^. Studies from various LMIC settings have found that ultrasound has a positive impact on patient management, safety, and task shifting^6,11^. Yet, ultrasound is neither widely available nor routinely used in the majority of LMIC^16^.

Multiple surveys have been undertaken to assess barriers to ultrasound use; however, most surveys have focused on settings in HIC. Common barriers have included lack of access to ultrasound education and training, to trained personnel, and to ultrasound equipment^17–23^. A previous 2015 survey specifically assessing perceived barriers to ultrasound use in LMIC settings found a lack of training (60%), cost of maintaining/obtaining/updating machines (50%), lack of reliable ultrasound maintenance capability (47%), and lack of equipment (46%) to be the most common barriers^4^. To better understand the current barriers and facilitators to ultrasound use in LMIC, we conducted a global online survey.

## Methods

After reviewing the peer-reviewed and published scientific literature to identify common ultrasound use cases and barriers, we developed and piloted a 42-question anonymous online survey in REDCap, separated into five sections, consisting of respondent and facility demographics, ultrasound training, ultrasound use, barriers and facilitators, and perceived importance of ultrasound (Appendix S1). Survey respondent and facility demographics included country, respondent education, current role(s), and information on healthcare facilities. Information on number, type, and duration of ultrasound training programs, certifications, and confidence with ultrasound use was also collected. The ultrasound use section of the survey focused on the respondent’s access, frequency of use, and types of ultrasound examinations and procedures conducted. Potential barriers and facilitators to ultrasound use were surveyed using Likert scales ranging from “definitely a barrier” to “definitely a facilitator.” Respondents were given the opportunity to add barriers and/or facilitators that were not listed in the survey. The final section of the survey asked respondents their opinions regarding the importance and value of ultrasound in relation to quality of care and patient outcomes as well as any fears or reservations they might have.

Multiple strategies were employed to target potential survey respondents. By email, we sent the online survey link to colleagues working and training in ultrasound in LMIC. We also contacted corresponding authors of publications identified during our multiple literature reviews, focusing on point-of-care ultrasound use, research, and barriers to use in LMIC (specifically, by country) and different types of ultrasound use cases (e.g., chest, abdominal, obstetric, etc.) in LMIC. Additionally, we contacted potential LMIC respondents via professional networks, online searches, ultrasound interest groups, and ultrasound training organizations operating in LMIC, who in turn distributed our survey link to their various memberships and listservs. Over 2000 potential participants were directly emailed by the study investigators with an unknown number of additional requests to participate sent through different organization memberships, listservs, phone conferences, online meetings, and tweets. To improve the response rate, all survey respondents were requested to forward the survey link to colleagues with ultrasound knowledge and/or experience in LMIC. We chose to also include respondents from HIC who had experience with ultrasound training and use in LMIC; when answering questions, respondents were instructed to focus on their experience with and use of ultrasound in LMIC. We used the World Bank Atlas method designations for LMIC vs HIC^24^.

The survey responses were analyzed descriptively in R with simple statistics. Exact binomial tests were used to assess whether the percent of respondents who judged items as either a barrier or facilitator were the same (50%, excluding “neither barrier nor facilitator” responses). No adjustments were made for multiple comparisons. The study and the waiver of informed consent was approved by the Hamilton Integrated Research Ethics Board, McMaster University, Ontario, Canada. All methods were performed in accordance with the World Medical Association’s Declaration of Helsinki.

## Results

**Figure 1:**
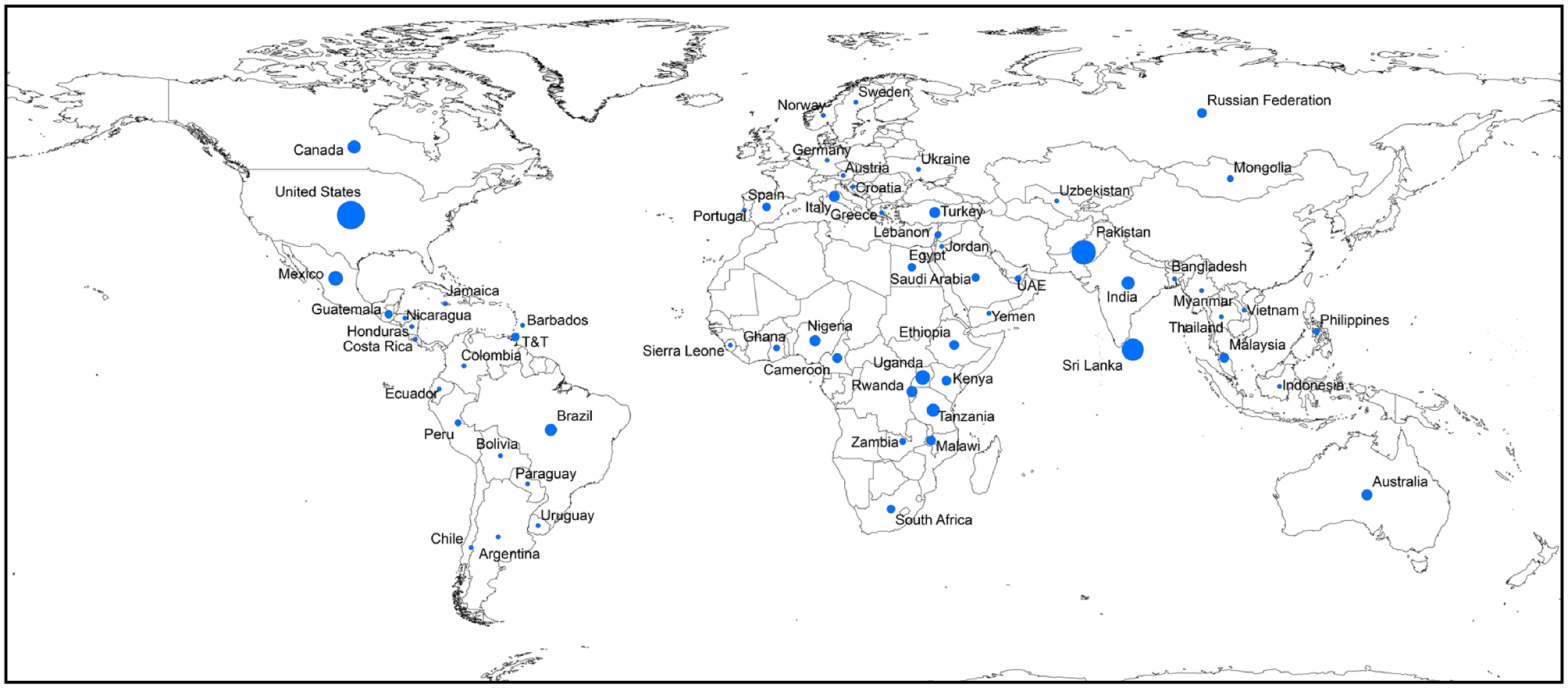
Survey respondents by country. Map created using ArcMap 10.7.1 (ESRI 2019. ArcGIS Desktop: Release 10.7.1. Redlands, CA: Environmental Systems Research Institute.).

A total of 241 respondents participated in this online survey, representing 62 countries (Fig. 1), with 70% from LMIC (Table 1). The mean age of respondents reporting their age was 43 (range 21 to 81) years and 48% of respondents reporting their sex were female. Of those reporting their highest level of education/training, 82% identified as physicians. Among respondents describing their facility, 89% worked in hospitals, 88% at a tertiary or academic hospital. More survey respondents worked in facilities located in an urban setting (83%), than in rural (10%) and peri-urban (9%) settings. More respondents worked in a publicly-funded facility (62%) than a privately-funded facility (21%), and 12% worked in both.

**Table 1:** Survey respondent and facility characteristics.

**Age** (N = 231)	
Mean (standard deviation), minimum, maximum	43.4 (10.9), 21, 81

	N (%)
**Sex** (N = 235)	
Female	113 (48.1)
Male	122 (51.9)
**Country **(N = 233)	
Low- or middle-income country	162 (69.5)
High-income country	71 (30.5)
**Highest education level** (N = 234)	
Medical officer/physician	192 (82.1)
Clinical officer	10 (4.3)
Master/PhD	10 (4.3)
Nurse/midwife	6 (2.6)
Medical assistant	5 (2.1)
Imaging technician	4 (1.7)
Administrator	2 (0.9)
Community health worker	0 (0.0)
Other education	5 (2.1)
Current role(s)* (N = 230)	
Medical officer/physician	163 (79.5)
Professor/lecturer/teacher	68 (33.2)
Research manager/coordinator	16 (7.8)
Clinical officer	12 (5.9)
Hospital administrator	7 (3.4)
Medical assistant	7 (3.4)
Research assistant	6 (2.9)
Ultrasound technician	6 (2.9)
Nurse	3 (1.5)
Community health worker	1 (0.5)
Midwife	0 (0.0)
Other role	11 (5.4)
Facility type(s)* (N = 224)	
Hospital	199 (88.8)
Health center	15 (6.7)
Office or clinic	11 (4.9)
Diagnostic imaging center	3 (1.3)
Community health outpost	2 (0.9)
Other facility type	16 (7.1)
Hospital type(s)* (N = 197)	
Tertiary or academic	173 (87.8)
Provincial	18 (9.1)
District	20 (10.2)
Sub-district	2 (1.0)
Other hospital type	4 (2.0)
Facility location(s)* (N = 224)	
Urban	187 (83.5)
Peri-urban	21 (9.4)
Rural	23 (10.3)
Other location	2 (0.9)
Facility funding source(s)* (N = 221)	
Public	138 (62.4)
Private	46 (20.8)
Public and private	26 (11.8)
Other source of funding	10 (8.3)

*Total percentage for individual question may be over 100% due to “Check all that apply” option for that question on the survey. The value for N provided for “Check all that apply” questions represents the number of respondents.

Regarding ultrasound education and/or training, 22% reported receiving no education and/or training and 44% of those noted they were self-taught (Table 2). Based on write-in responses, those who were self-taught relied on online courses, YouTube tutorials, papers, and books as resources. Of those receiving at least one training, 75% reported having participated in more than one training program with a mean of 3 (range 1 to 7 or more) trainings. The most common types of trainings included continuing medical education (36%), training during school (22%), attending seminars during in-person conferences (18%), and participating in online courses (11%). The median duration of the first training was 3 days. Of those reporting having received training, 65% received a certificate. Among those who detailed how long ago their most recent training was completed, 42% reported their last ultrasound training between 1 and 5 years ago, 34% less than 1 year ago, and 24% more than 5 years ago. After completion of their most recent training, 90% felt somewhat confident, confident, or very confident in their ability to conduct ultrasounds. Almost two-thirds of respondents reported training others on the use of ultrasound. Upon asking respondents to rate their ultrasound expertise, 45% noted they were somewhat experienced, 25% reported being inexperienced or somewhat inexperienced, 20% identified as experts, and 10% reported no experience.

**Table 2:** Ultrasound training.

	N (%)
**Ultrasound training **(N = 198)	
Yes	154 (77.8)
No	44 (22.2)
**Self-taught in ultrasound use** (N = 43)	
Yes	19 (44.2)
No	24 (55.8)
**Number of ultrasound trainings completed** (N = 148)	
1	37 (25.0)
2	41 (27.7)
3	27 (18.2)
4	12 (8.1)
5	4 (2.7)
6	1 (0.7)
7 or more	26 (17.6)
Type(s) of ultrasound training(s)* (N = 145)	
Continued medical education ultrasound training course	161 (36.3)
Training occurred during schooling	98 (22.1)
Seminar during in-person conference	78 (17.6)
Online course	48 (10.8)
As part of a study/research project	40 (9.0)
Other type of training	22 (5.0)
**Certification during ultrasound training(s) **(N = 144)	
Yes	93 (64.6)
No	51 (35.4)
**Most recent ultrasound training** (N = 134)	
0–1 year	46 (34.3)
1–5 years	56 (41.8)
> 5 years	32 (23.9)
**Overall ultrasound training confidence** (N = 145)	
Very confident	31 (21.4)
Confident	63 (43.5)
Somewhat confident	37 (25.5)
Not confident	7 (4.8)
Not sure	7 (4.8)
**Ultrasound expertise** (N = 185)	
Very experienced/expert	37(20.0)
Somewhat experienced	83 (44.9)
Somewhat inexperienced	20 (10.8)
Inexperienced/novice	26 (14.1)
No experience	19(10.3)
**Train others on the use of ultrasound **(N = 183)	
Yes	111 (60.7)
No	72 (39.3)

*Total percentage for individual question may be over 100% due to “Check all that apply” option for that question on the survey. The value for N provided for “Check all that apply” questions represents the number of respondents.

Access to ultrasound in their current clinical setting was reported by 87.5% respondents and 12.5% reported no access to ultrasound (Table 3). Portable ultrasounds only were used by 51%, non-portable ultrasounds only were used by 16%, and 33% used both. Probes used included linear (82%), curved (76%), phased-array (45%), multi-use (21%), and endocavitary (16%). When asked about frequency of use, 46% reported daily ultrasound use, 26% weekly use, 11% monthly or every few months use, and 17% never or rare use. Respondents with more ultrasound experience reported more frequent ultrasound use. Among expert users, 79% reported daily use vs 14% with weekly use. Among somewhat experienced users, 57% reported daily use vs 28% with weekly use. Among inexperienced or somewhat inexperienced users, 13% reported daily use, 30% weekly use, 22% monthly or every few months use, and 35% reported never or rare use (data not shown).

**Table 3:** Ultrasound access and use in low- and middle-income countries.

	N (%)
**Ultrasound access and use in current setting** (N = 184)	
Access	161 (87.5)
No access	23 (12.5)

**Number of ultrasound machines** (N = 154)	
Mean (standard deviation), minimum, median, maximum	2.9 (4.6), 1, 2, 50

	N (%)
**Type(s) of ultrasound machine(s)** (N = 160)	
Portable	82 (51.3)
Not portable	25 (15.6)
Both portable and not portable	53 (33.1)
**Handheld portable ultrasound machine** (N = 134)	
Yes	68 (50.8)
No	66 (49.3)
Type(s) of ultrasound probe(s)* (N = 157)	
Linear	129 (82.2)
Curved	119 (75.8)
Phased array	70 (44.6)
Multi-use probe	33 (21.0)
Endocavitary	25 (15.9)
Other type of probe	6 (3.8)
**Ultrasound use frequency** (N = 183)	
Daily	84 (45.9)
Weekly	47 (25.7)
Monthly	11 (6.0)
Every few months	10 (5.5)
Rarely	14 (7.7)
Never	17 (9.3)
Ultrasound use indication(s)* (N = 177)	
IV insertion	109 (61.6)
Lung	103 (58.2)
Cardiac/echocardiography	98 (55.4)
Trauma/focused assessment for trauma (FAST)	76 (42.9)
Thoracentesis	70 (39.6)
Paracentesis	58 (32.8)
Kidney and/or bladder	52 (29.4)
Skin and soft tissue	47 (26.6)
Gallbladder	46 (26.0)
Pericardiocentesis	42 (23.7)
Obstetrics and/or gynecology	40 (22.6)
Nerve block	39 (22.0)
Musculoskeletal	32 (18.1)
Abdominal aortic aneurysm	24 (13.6)
Other indication	31 (17.5)
**Workplace policies/guidelines** (N = 157)	
Yes	53 (33.8)
No	104 (66.2)

*Total percentage for individual question may be over 100% due to “Check all that apply” option for that question on the survey. The value for N provided for “Check all that apply” questions represents the number of respondents.

Ultrasound was most commonly used for procedures, including intravenous line placement (62%), thoracentesis (40%), paracentesis (33%), pericardiocentesis (24%), and nerve blocks (22%) (Table 3). The most common diagnostic uses of ultrasound included evaluations of the lung (58%), heart/echocardiography (55%) and focused assessment for trauma (43%). Other uses included obstetrics (23%), kidney/bladder (29%), skin and soft tissue (27%), gallbladder (26%), musculoskeletal (18%), and abdominal aortic aneurysm (14%) examinations. The type of ultrasound use most commonly conducted by respondents did not change when stratified by country income status. Only 34% noted their place of work had policies and/or guidelines for ultrasound use.

**Figure 2:**
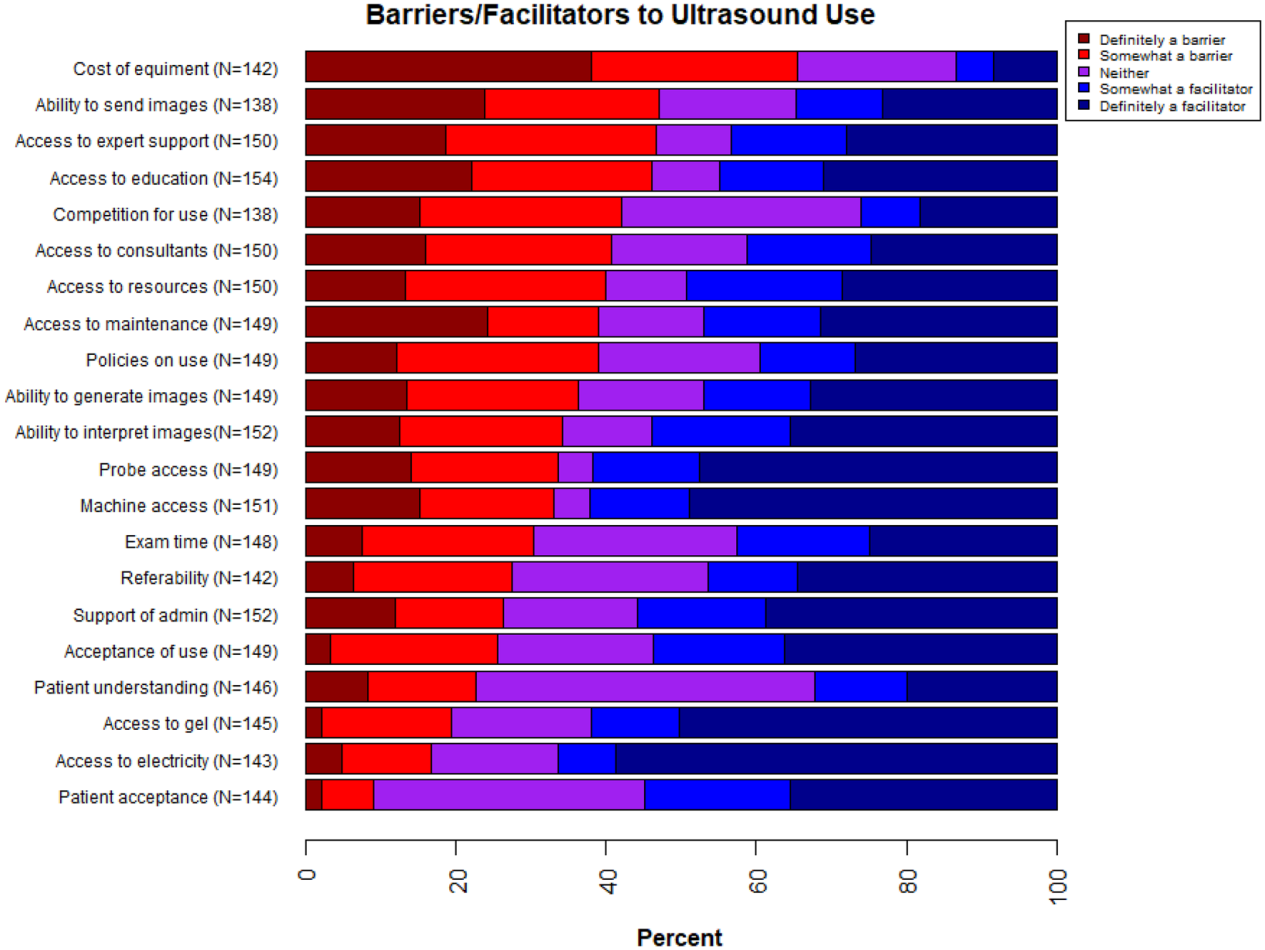
Barriers and facilitators to ultrasound use in low- and middle-income countries.

When asked to identify the top barrier and the top facilitator among 21 prespecified potential barriers/facilitators, the top barrier to ultrasound use was around access: access to an ultrasound machine (17%); access to support from an ultrasound expert for ongoing mentoring and training (14%); access to ultrasound education and/or training (12%); access to maintenance and repair for ultrasound machines and/or probes (10%); and cost of ultrasound machines, probes, and equipment (9%) (Table 4). The top facilitator to ultrasound use was also around access to an ultrasound machine (53%). When asked to rate the pre-specified 21 potential barriers/facilitators, nine were significantly more often listed as a facilitator (somewhat or definitely) than a barrier (excluding neither a barrier nor a facilitator responses). One of the nine was access to an ultrasound machine, with 65% reporting access to an ultrasound machine as a facilitator and 35% as a barrier (Table 4; Fig. 2). Similarly, access to an ultrasound probe, ultrasound gel, and reliable electricity/power were more often seen as a facilitator than as a barrier. Also more often seen as facilitators than barriers were healthcare providers’ acceptance and hospital administration’s support of ultrasound as an appropriate imaging tool, provider ability to interpret ultrasound images, ability to refer patients to a higher level of care for management of ultrasound findings, and patient acceptance of having ultrasound performed on them. More often viewed as barriers than facilitators were cost of the ultrasound machine, probe, or equipment (83% as barrier; 17% as facilitator), and competition for use with other providers and/or departments (62% as barrier; 38% as facilitator).

**Table 4:** Barriers and facilitators to ultrasound use in low- and middle-income countries.

	Evaluation of individual potential barriers and facilitators						p-value*	Top choice barrier or facilitator	
	Definitely a barrier N (%)	Somewhat a barrier N (%)	Neither barrier nor facilitator N (%)	Somewhat a facilitator N (%)	Definitely a facilitator N (%)	Not applicable N (%)		Top barrier**N (%) out of 162	Top facilitator**N (%) out of 159
Cost of equipment (N = 158)	54 (34.2)	39 (24.7)	30 (19.0)	7 (4.4)	12 (7.6)	16 (10.1)	** < 0.001**	14 (8.6)	1 (0.6)
Ability to send images for remote expert interpretation (N = 159)	33 (20.8)	32 (20.1)	25 (15.7)	16 (10.1)	32 (20.1)	21 (13.2)	0.13	2 (1.2)	2 (1.3)
Access to expert support for ongoing mentoring/training (N = 159)	28 (17.6)	42 (26.4)	15 (9.4)	23 (14.5)	42 (26.4)	9 (5.7)	0.73	22 (13.6)	6 (3.8)
Access to education/training (N = 161)	34 (21.1)	37 (23.0)	14 (8.7)	21 (13.0)	48 (29.8)	7 (4.4)	0.93	20 (12.4)	11 (6.9)
Competition for use (N = 159)	21 (13.2)	37 (23.3)	44 (27.7)	11 (6.9)	25 (15.7)	21 (13.2)	**0.03**	6 (3.7)	0 (0.00)
Access to radiology consultants (N = 160)	24 (15.0)	37 (23.1)	27 (16.9)	25 (15.6)	37 (23.1)	10 (6.3)	1.0	1 (0.6)	4 (2.5)
Access to learning resources (N = 158)	20 (12.7)	40 (25.3)	16 (10.1)	31 (19.6)	43 (27.2)	8 (5.1)	0.26	4 (2.5)	5 (3.1)
Access to maintenance/repair (N = 160)	36 (22.5)	22 (13.8)	21 (13.1)	23 (14.4)	47 (29.4)	11 (6.9)	0.33	16 (9.9)	0 (0.00)
Policies/guidelines on use (N = 160)	18 (11.6)	40 (25.0)	32 (20.0)	19 (11.9)	40 (25.0)	11 (6.9)	1.0	5 (3.1)	4 (2.5)
Ability to generate high-quality images (N = 157)	20 (12.7)	34 (21.7)	25 (15.9)	21 (13.4)	49 (31.2)	8 (5.1)	0.18	6 (3.7)	2 (1.3)
Ability to interpret images (N = 160)	19 (11.9)	33 (20.6)	18 (11.3)	28 (17.5)	54 (33.8)	8 (5.0)	**0.012**	2 (1.2)	4 (2.5)
Access to probes (N = 161)	21 (13.0)	29 (18.0)	7 (4.4)	21 (13.0)	71 (44.1)	12 (7.5)	** < 0.001**	4 (2.5)	1 (0.6)
Access to machines (N = 160)	23 (14.4)	27 (16.9)	7 (4.4)	20 (12.5)	74 (46.3)	9 (5.6)	** < 0.001**	27 (16.7)	84 (52.8)
Exam time (N = 159)	11 (6.9)	34 (21.4)	40 (25.2)	26 (16.4)	37 (23.3)	11 (6.9)	0.10	2 (1.2)	2 (1.3)
Ability to refer based on ultrasound findings (N = 160)	9 (5.6)	30 (18.8)	37 (23.1)	17 (10.6)	49 (30.6)	18 (11.3)	**0.011**	2 (1.2)	2 (1.3)
Support of use by hospital administration (N = 159)	18 (11.3)	22 (13.8)	27 (17.0)	26 (16.4)	59 (37.1)	7 (4.4)	** < 0.001**	4 (2.5)	7 (4.4)
Acceptance of use by healthcare providers (N = 159)	5 (3.1)	33 (20.75)	31 (19.5)	26 (16.4)	54 (34.0)	10 (6.3)	** < 0.001**	6 (3.7)	7 (4.4)
Patient understanding (N = 159)	12 (7.6)	21 (13.2)	66 (41.5)	18 (11.3)	29 (18.2)	13 (8.2)	0.15	1 (0.6)	0 (0.0)
Access to gel (N = 159)	3 (1.9)	25 (15.7)	27 (17.0)	17 (10.7)	73 (45.9)	14 (8.8)	** < 0.001**	0 (0.0)	2 (1.3)
Access to electricity/power (N = 160)	7 (4.4)	17 (10.6)	24 (15.0)	11 (6.9)	84 (52.5)	17 (10.6)	** < 0.001**	0 (0.0)	4 (2.5)
Acceptance of use by patients (N = 161)	3 (1.9)	10 (6.2)	52 (32.3)	28 (17.4)	51 (31.7)	17 (10.6)	** < 0.001**	0 (0.0)	3 (1.9)

*p-value comes from combining definitely a barrier with somewhat a barrier and combining definitely a facilitator with somewhat a facilitator for each row, leaving out neither barrier nor facilitator and not applicable, and performing an exact binomial test to determine whether the proportion of barriers for each row is different from 0.50. Significant values are in bold.

**N reported includes not applicable (N/A) responses. For top barrier, 14 respondents answered N/A; for top facilitator, 7 respondents answered N/A.

Respondents were more split over access to maintenance and repair for ultrasound machines and/or probes, access to ultrasound education and/or training, access to ultrasound learning resources, access to support from an ultrasound expert for ongoing mentoring and training, access to radiology consultants for ultrasound image acquisition and/or interpretation, ability to send ultrasound images for interpretation by a remote ultrasound expert, ability to generate high-quality images, amount of time it takes to conduct an ultrasound exam, patient understanding of ultrasound technology, and policies and/or guidelines on ultrasound use (Table 4; Fig. 2). When access to an ultrasound machine or probe was cited as a barrier, the main reasons were unavailability of ultrasound machines (53%); unavailability of probes (51%); and competition for use by other departments (38%) (Table 5).

**Table 5:** Respondent opinions on ultrasound use in low- and middle-income countries.

	N (%)
Reason(s) for ultrasound access being viewed as a barrier* (N = 53)	
Ultrasound machine unavailable	28 (52.8)
Ultrasound probe unavailable	27 (50.9)
Competition for use by different departments	20 (37.7)
Competition for use in the same department	14 (26.4)
Other reason	7 (13.2)
**Location of ultrasound expert if access to support from an ultrasound expert for ongoing mentoring/training is viewed as a facilitator** (N = 60)	
Local	50 (83.3)
Remote	10 (16.7)
**Importance of having ultrasound access** (N = 162)	
Very important	114 (70.4)
Important	16 (9.9)
Neither important nor unimportant	1 (0.6)
Unimportant	0 (0.0)
Very unimportant	31 (19.1)
**Ultrasound improves quality of care** (N = 163)	
Strongly agree	134 (82.2)
Agree	23 (14.1)
Neither agree nor disagree	0 (0.0)
Disagree	0 (0.0)
Strongly disagree	6 (3.7)
**Ultrasound improves patient outcomes** (N = 163)	
Strongly agree	105 (64.4)
Agree	51 (31.3)
Neither agree nor disagree	4 (2.5)
Disagree	0 (0.0)
Strongly disagree	3 (1.8)
**Fear(s)/reservation(s) associated with ultrasound use** (N = 163)	
Yes	30 (18.4)
No	133 (81.6)
Types of fear(s)/reservation(s) associated with ultrasound use* (N = 28)	
Misdiagnosis	20 (71.4)
Malpractice	5 (17.9)
Leadership/administration not supporting use	5 (17.9)
Peers not supporting use	4 (14.3)
Patients not understanding technology	3 (10.7)
Other fear/reservation	5 (17.9)

*Total percentage for individual question may be over 100% due to “Check all that apply” option for that question on the survey. The value for N provided for “Check all that apply” questions represents the number of respondents.

The majority of respondents (80%) noted that access to ultrasound was important or very important (Table 5). Among respondents, 96% either strongly agreed or agreed that ultrasound improves quality of care and patient outcomes. When queried about any fears or reservations associated with using ultrasound, the majority (82%) said they had none. Of those expressing fears or reservations, misdiagnosis (71%) was the most common.

## Discussion

Informed by 241 respondents from 62 countries, 70% reporting from LMIC and 30% from HIC reporting about experience in LMIC, our global online survey revealed that both the single biggest barrier and the single biggest facilitator to ultrasound use in LMIC was access to an ultrasound machine. Access remains a dominant theme for ultrasound, as it does for most global health technologies in LMIC^25^. Access to ultrasound equipment, including machines, probes, and gel, and access to reliable electricity and/or power were the most commonly reported facilitators to ultrasound use in our survey. Notably, the majority of respondents reported that ultrasound is very important for patient care, agreeing or strongly agreeing that ultrasound improves both quality of care and patient outcomes.

Based on previous ultrasound research and other health technology work assessing barriers to use in LMIC, our pre-survey hypothesis was that perceived cost and access to training likely would be the biggest barriers. While access to an ultrasound machine was cited more frequently as the single biggest barrier, cost of equipment was more frequently noted as a barrier when considering 21 potential barriers/facilitators. Access to support from an ultrasound expert for ongoing mentoring and training and access to ultrasound education and/or training were also frequently cited as barriers. As ultrasound education and training of LMIC healthcare providers have increased, it remains unclear what constitutes optimal training, including type, scope, duration, frequency, and quality assurance, among other factors^8,11,26–34^.

Evaluation of ultrasound use, particularly in LMIC, has focused most commonly on obstetrical applications as well as cardiac, lung, and trauma, among others^3–12^. With the advent of the COVID-19 pandemic, we have witnessed increased interest and literature around the role of point-of-care ultrasound^35,36^. Use cases with special relevance to LMIC settings include diagnosis and management of infectious diseases such as malaria, tuberculosis, dengue, schistosomiasis, leishmaniasis, and cystic echinococcosis^11,37–45^. In our survey, respondents reported using ultrasound most frequently for procedures; other common applications included evaluations on the lungs, heart/echocardiography, and focused assessments for trauma. Because we did not specifically target obstetricians or midwives, our sample, consisting primarily of physicians at tertiary hospitals using portable ultrasounds, may contribute to why bedside procedures and examinations were more commonly reported applications than obstetrical evaluations. Other surveys specifically targeting midwives, nurses, and obstetricians demonstrate obstetrics as a more common use case^11,26^.

While use of ultrasound in patient care was viewed favorably by the overwhelming majority of survey respondents, a minority of respondents did note some reservation, specifically regarding fear of potential misdiagnosis. A study evaluating the feasibility, usability, and acceptability of lung ultrasound for the diagnosis of pediatric pneumonia among healthcare providers and caregivers in Mozambique and Pakistan also demonstrated an overall positive attitude toward ultrasound with few exceptions^46^. Interviewed healthcare providers and caregivers favored ultrasound for its rapid results, lack of ionizing radiation, and potential for improved diagnostic accuracy and streamlined workflow. Healthcare providers believed ultrasound could be successfully integrated into their healthcare setting with sufficient training, knowledge sharing, policymaker buy-in, and patient acceptance.

Despite distributing our survey widely, the small number of respondents completing the survey was a limitation and may be subject to sampling bias. In addition, not all respondents completed answers to all survey questions. Further limiting the generalizability, the large majority of our respondents were physicians with training in ultrasound who reported working at tertiary hospitals in urban settings. Our respondents included relatively small numbers of other classes of healthcare providers or those from other levels of the healthcare system. Given that we were asking about the barriers and facilitators to ultrasound use, we targeted healthcare providers who had some experience with ultrasound use and this was reflected by the finding that most respondents had received formal ultrasound training, with a majority having participated in more than one training. This may also help explain why our respondents were split in reporting access to ultrasound education, training, ongoing mentoring and learning resources as a barrier vs a facilitator.

## Conclusions

A versatile and safe imaging technology with a wide variety of applications and use cases, ultrasound has the potential to revolutionize the ability of providers in LMIC settings to dynamically see inside the body at point-of-care to help inform assessment, diagnosis, and management of illness. However, to realize that potential, an understanding of barriers and facilitators to use in LMIC settings is important. Improving access to low-cost ultrasound equipment will be critical to increasing ultrasound use among those who are trained. It is likely that among those healthcare providers not yet familiar with or who have little to no experience with ultrasound, education and training will also be essential. Exciting and promising innovations such as less expensive, hand-held and artificial intelligence-assisted ultrasound technologies may address some of these barriers in LMIC, including but not limited to enabling ongoing education and training, enhanced navigation and quality-assured image acquisition, and more accurate and reliable measurement, assessment, and interpretation. Emerging and future technological advances in ultrasound hold the possibility of expanding access and transforming healthcare in LMIC with improved screening, prediction, triage, diagnosis, treatment, and monitoring of a large diversity of illnesses, diseases, and disorders as well as of growth, development, and function.

## Supplementary Information

Supplementary Information.

## Data Availability

Access to data will be provided to researchers subject to submission of a research proposal and signing a Data Use Agreement. Interested researchers can request access to the data by contacting the corresponding author.
