# Supplementary material for: A survey of barriers and facilitators to ultrasound use in low- and middle-income countries
Source: Sci Rep. 2023 Feb 27;13:3322. doi: 10.1038/s41598-023-30454-w (PMC9969046; doi:10.1038/s41598-023-30454-w)
Supplement: Supplementary file 1 — Supplementary Information. [file 41598_2023_30454_MOESM1_ESM.docx]

| **Appendix S1: Data Dictionary Codebook** |  |
| --- | --- |

 Collapse all instruments

| **#** | **Variable / Field Name** | **Field Label**  ***Field Note*** | **Field Attributes (Field Type, Validation, Choices, Calculations, etc.)** |
| --- | --- | --- | --- |
| Instrument:**Ultrasound Barriers & Facilitators**(ultrasound_barriers_facilitators) Enabled as survey Collapse | | | |
| 1 | [record_id] | Record ID | text |
| 2 | [introduction] | You are being invited to participate in this online research survey because you have knowledge of or experience with the use of ultrasound in low- and middle-income countries. We are interested in learning more about the barriers to and facilitators for ultrasound use in these settings. A survey of barriers and facilitators to ultrasound use in low-and middle-income countries. Survey questions will focus on respondent demographics, working location(s), ultrasound education and training, and use experience, including respondent opinions regarding specific ultrasound barriers and facilitators and the importance of ultrasound. The survey will be anonymous and no identifying information will be collected. You can choose to stop the survey at any time; however, once the survey is submitted, the responses cannot be removed.  We estimate the survey will take 5-10 minutes to complete. For any questions about the survey, please contact study investigators at ultrasound2022@yahoo.com. | descriptive |
| 3 | [age] | Section Header: *Respondent*  1. What is your age? (years)  *years* | text (number, Min: 18, Max: 150) |
| 4 | [sex] | 2. What is your sex? | radio   \| 1 \| Male \| \| --- \| --- \| \| 2 \| Female \| \| 3 \| Intersex or Non-binary \| |
| 5 | [country] | 3. What country do you live in? | dropdown   \| 1 \| Afghanistan \| \| --- \| --- \| \| 2 \| Albania \| \| 3 \| Algeria \| \| 4 \| Andorra \| \| 5 \| Angola \| \| 6 \| Antigua and Barbuda \| \| 7 \| Argentina \| \| 8 \| Armenia \| \| 9 \| Australia \| \| 10 \| Austria \| \| 11 \| Azerbaijan \| \| 12 \| The Bahamas \| \| 13 \| Bahrain \| \| 14 \| Bangladesh \| \| 15 \| Barbados \| \| 16 \| Belarus \| \| 17 \| Belgium \| \| 18 \| Belize \| \| 19 \| Benin \| \| 20 \| Bhutan \| \| 21 \| Bolivia \| \| 22 \| Bosnia and Herzegovina \| \| 23 \| Botswana \| \| 24 \| Brazil \| \| 25 \| Brunei \| \| 26 \| Bulgaria \| \| 27 \| Burkina Faso \| \| 28 \| Burundi \| \| 29 \| Cambodia \| \| 30 \| Cameroon \| \| 31 \| Canada \| \| 32 \| Cape Verde \| \| 33 \| Central African Republic \| \| 34 \| Chad \| \| 35 \| Chile \| \| 36 \| China \| \| 37 \| Colombia \| \| 38 \| Comoros \| \| 39 \| Republic of the Congo \| \| 40 \| Democratic Republic of the Congo \| \| 41 \| Costa Rica \| \| 42 \| Cote d'Ivoire \| \| 43 \| Croatia \| \| 44 \| Cuba \| \| 45 \| Cyprus \| \| 46 \| Czech Republic \| \| 47 \| Denmark \| \| 48 \| Djibouti \| \| 49 \| Dominica \| \| 50 \| Dominican Republic \| \| 51 \| East Timor (Timor-Leste) \| \| 52 \| Ecuador \| \| 53 \| Egypt \| \| 54 \| El Salvador \| \| 55 \| Equatorial Guinea \| \| 56 \| Eritrea \| \| 57 \| Estonia \| \| 58 \| Ethiopia \| \| 59 \| Fiji \| \| 60 \| Finland \| \| 61 \| France \| \| 62 \| Gabon \| \| 63 \| The Gambia \| \| 64 \| Georgia \| \| 65 \| Germany \| \| 66 \| Ghana \| \| 67 \| Greece \| \| 68 \| Grenada \| \| 69 \| Guatemala \| \| 70 \| Guinea \| \| 71 \| Guinea-Bissau \| \| 72 \| Guyana \| \| 73 \| Haiti \| \| 74 \| Honduras \| \| 75 \| Hungary \| \| 76 \| Iceland \| \| 77 \| India \| \| 78 \| Indonesia \| \| 79 \| Iran \| \| 80 \| Iraq \| \| 81 \| Ireland \| \| 82 \| Israel \| \| 83 \| Italy \| \| 84 \| Jamaica \| \| 85 \| Japan \| \| 86 \| Jordan \| \| 87 \| Kazakhstan \| \| 88 \| Kenya \| \| 89 \| Kiribati \| \| 90 \| North Korea \| \| 91 \| South Korea \| \| 92 \| Kosovo \| \| 93 \| Kuwait \| \| 94 \| Kyrgyzstan \| \| 95 \| Laos \| \| 96 \| Latvia \| \| 97 \| Lebanon \| \| 98 \| Lesotho \| \| 99 \| Liberia \| \| 100 \| Libya \| \| 101 \| Liechtenstein \| \| 102 \| Lithuania \| \| 103 \| Luxembourg \| \| 104 \| Macedonia \| \| 105 \| Madagascar \| \| 106 \| Malawi \| \| 107 \| Malaysia \| \| 108 \| Maldives \| \| 109 \| Mali \| \| 110 \| Malta \| \| 111 \| Marshall Islands \| \| 112 \| Mauritania \| \| 113 \| Mauritius \| \| 114 \| Mexico \| \| Micronesia \| Federated States of \| \| 115 \| Moldova \| \| 116 \| Monaco \| \| 117 \| Mongolia \| \| 118 \| Montenegro \| \| 119 \| Morocco \| \| 120 \| Mozambique \| \| 121 \| Myanmar (Burma) \| \| 122 \| Namibia \| \| 123 \| Nauru \| \| 124 \| Nepal \| \| 125 \| Netherlands \| \| 126 \| New Zealand \| \| 127 \| Nicaragua \| \| 128 \| Niger \| \| 129 \| Nigeria \| \| 130 \| Norway \| \| 131 \| Oman \| \| 132 \| Pakistan \| \| 133 \| Palau \| \| 134 \| Panama \| \| 135 \| Papua New Guinea \| \| 136 \| Paraguay \| \| 137 \| Peru \| \| 138 \| Philippines \| \| 139 \| Poland \| \| 140 \| Portugal \| \| 141 \| Qatar \| \| 142 \| Romania \| \| 143 \| Russia \| \| 144 \| Rwanda \| \| 145 \| Saint Kitts and Nevis \| \| 146 \| Saint Lucia \| \| 147 \| Saint Vincent and the Grenadines \| \| 148 \| Samoa \| \| 149 \| San Marino \| \| 150 \| Sao Tome and Principe \| \| 151 \| Saudi Arabia \| \| 152 \| Senegal \| \| 153 \| Serbia \| \| 154 \| Seychelles \| \| 155 \| Sierra Leone \| \| 156 \| Singapore \| \| 157 \| Slovakia \| \| 158 \| Slovenia \| \| 159 \| Solomon Islands \| \| 160 \| Somalia \| \| 161 \| South Africa \| \| 162 \| South Sudan \| \| 163 \| Spain \| \| 164 \| Sri Lanka \| \| 165 \| Sudan \| \| 166 \| Suriname \| \| 167 \| Swaziland \| \| 168 \| Sweden \| \| 169 \| Switzerland \| \| 170 \| Syria \| \| 171 \| Taiwan \| \| 172 \| Tajikistan \| \| 173 \| Tanzania \| \| 174 \| Thailand \| \| 175 \| Togo \| \| 176 \| Tonga \| \| 177 \| Trinidad and Tobago \| \| 178 \| Tunisia \| \| 179 \| Turkey \| \| 180 \| Turkmenistan \| \| 181 \| Tuvalu \| \| 182 \| Uganda \| \| 183 \| Ukraine \| \| 184 \| United Arab Emirates \| \| 185 \| United Kingdom \| \| 186 \| United States of America \| \| 187 \| Uruguay \| \| 188 \| Uzbekistan \| \| 189 \| Vanuatu \| \| 190 \| Vatican City (Holy See) \| \| 191 \| Venezuela \| \| 192 \| Vietnam \| \| 193 \| Yemen \| \| 194 \| Zambia \| \| 195 \| Zimbabwe \| |
| 6 | [education] | 4. What is your highest level of education/training? | dropdown   \| 1 \| Administrator \| \| --- \| --- \| \| 2 \| Medical officer/ Physician \| \| 3 \| Clinical officer \| \| 4 \| Nurse \| \| 5 \| Midwife \| \| 6 \| Ultrasound technician \| \| 7 \| Medical assistant \| \| 8 \| Community health worker \| \| 9 \| Other (Please specify) \| |
| 7 | [education_other]  Show the field ONLY if:  [education] = '9' | Other education (please specify):  *Other education* | notes |
| 8 | [currentrole] | 5. What is your current role? (Please check all that apply) | checkbox   \| 1 \| currentrole___1 \| Hospital administrator \| \| --- \| --- \| --- \| \| 2 \| currentrole___2 \| Medical Officer/Physician \| \| 3 \| currentrole___3 \| Clinical officer \| \| 4 \| currentrole___4 \| Nurse \| \| 5 \| currentrole___5 \| Midwife \| \| 6 \| currentrole___6 \| Ultrasound technician \| \| 7 \| currentrole___7 \| Medical assistant \| \| 8 \| currentrole___8 \| Community health worker \| \| 9 \| currentrole___9 \| Professor/Lecturer/Teacher \| \| 10 \| currentrole___10 \| Research manager/coordinator \| \| 11 \| currentrole___11 \| Research assistant \| \| 12 \| currentrole___12 \| Other (Please specify) \| |
| 9 | [currentrole_other]  Show the field ONLY if:  [currentrole(12)]=1 | Other role (Please specify):  *Other role* | text |
| 10 | [extra_comments_intro] | Please feel free to provide any additional comments or explanations to any of the above questions and/or responses. | notes |
| 11 | [facility] | Section Header: *Facility (12% Completed)*          6. What type of facility do you work in? (Please check all that apply) | checkbox   \| 1 \| facility___1 \| Hospital (Please specify level) \| \| --- \| --- \| --- \| \| 2 \| facility___2 \| Health center \| \| 3 \| facility___3 \| Office or clinic \| \| 4 \| facility___4 \| Diagnostic imaging center \| \| 5 \| facility___5 \| Community health outpost \| \| 6 \| facility___6 \| Other (Please specify) \| |
| 12 | [facility_hospital]  Show the field ONLY if:  [facility(1)]=1 | Hospital (Please specify): | checkbox   \| 1 \| facility_hospital___1 \| Tertiary or Academic \| \| --- \| --- \| --- \| \| 2 \| facility_hospital___2 \| Provincial \| \| 3 \| facility_hospital___3 \| District \| \| 4 \| facility_hospital___4 \| Sub-district \| \| 5 \| facility_hospital___5 \| Other (Please specify) \| |
| 13 | [facility_hospital_other]  Show the field ONLY if:  [facility_hospital(5)]=1 | Other hospital (Please specify):  *Other hospital* | text |
| 14 | [facility_other]  Show the field ONLY if:  [facility(6)]=1 | Other facility not listed above (Please specify):  *Other facility* | text |
| 15 | [facilitylocation] | 7. Where is your facility located? (Please check all that apply) | checkbox   \| 1 \| facilitylocation___1 \| Urban \| \| --- \| --- \| --- \| \| 2 \| facilitylocation___2 \| Peri-urban \| \| 3 \| facilitylocation___3 \| Rural \| \| 4 \| facilitylocation___4 \| Other (Please specify) \| |
| 16 | [facilitylocation_other]  Show the field ONLY if:  [facilitylocation(4)]=1 | Other location (Please specify):  *Other location* | text |
| 17 | [facilityfunded] | 8. Do you work in a publicly- or privately-funded facility? (Please check all that apply) | checkbox   \| 1 \| facilityfunded___1 \| Public \| \| --- \| --- \| --- \| \| 2 \| facilityfunded___2 \| Private \| \| 3 \| facilityfunded___3 \| Other funding source \| |
| 18 | [facilityfunded_other]  Show the field ONLY if:  [facilityfunded(3)]=1 | Other funding source (Please specify):  *Other funding source* | text |
| 19 | [extra_comments_training] | Please feel free to provide any additional comments or explanations to any of the above questions and/or responses. | notes |
| 20 | [ultrasoundtraining] | Section Header: *Ultrasound Training (20% Completed)*    9. Have you had any education and/or training on ultrasound? | radio   \| 1 \| Yes \| \| --- \| --- \| \| 2 \| No \| |
| 21 | [training_number]  Show the field ONLY if:  [ultrasoundtraining]=1 | a. How many trainings have you completed? | radio   \| 1 \| 1 \| \| --- \| --- \| \| 2 \| 2 \| \| 3 \| 3 \| \| 4 \| 4 \| \| 5 \| 5 \| \| 6 \| 6 \| \| 7 \| 7 or more \| |
| 22 | [training_type_1]  Show the field ONLY if:  [training_number]=1 OR [training_number]=2 OR [training_number]=3 OR [training_number]=4 OR [training_number]=5 OR [training_number]=6 OR [training_number]=7 | 9-1b. For your first training, what type of training did you complete? (Please check all that apply) | checkbox   \| 1 \| training_type_1___1 \| Training occurred during schooling \| \| --- \| --- \| --- \| \| 2 \| training_type_1___2 \| Continuing medical education (CME) ultrasound training course \| \| 3 \| training_type_1___3 \| Seminar during in-person conference \| \| 4 \| training_type_1___4 \| Online course \| \| 5 \| training_type_1___5 \| As part of a study/research project \| \| 6 \| training_type_1___6 \| Other (Please specify) \| |
| 23 | [first_training_type_other]  Show the field ONLY if:  [training_type_1(6)]=1 | Other (Please specify):  *Other type* | text |
| 24 | [training_days_1]  Show the field ONLY if:  [training_number]=1 OR [training_number]=2 OR [training_number]=3 OR [training_number]=4 OR [training_number]=5 OR [training_number]=6 OR [training_number]=7 | 9-1c. For your first training, how long was the training? (days)  *days* | text (number) |
| 25 | [training_certification_1]  Show the field ONLY if:  [training_number]=1 OR [training_number]=2 OR [training_number]=3 OR [training_number]=4 OR [training_number]=5 OR [training_number]=6 OR [training_number]=7 | 9-1d. For your first training, did you receive some sort of certification? | radio   \| 1 \| Yes \| \| --- \| --- \| \| 2 \| No \| |
| 26 | [training_organization_1]  Show the field ONLY if:  [training_number]=1 OR [training_number]=2 OR [training_number]=3 OR [training_number]=4 OR [training_number]=5 OR [training_number]=6 OR [training_number]=7 | 9-1e. For your first training, what organization or who conducted the training?  *Organization* | text |
| 27 | [training_type_2]  Show the field ONLY if:  [training_number]=2 OR [training_number]=3 OR [training_number]=4 OR [training_number]=5 OR [training_number]=6 OR [training_number]=7 | 9-2b. For your second training, what type of training did you complete?  (Please check all that apply) | checkbox   \| 1 \| training_type_2___1 \| Training occurred during schooling \| \| --- \| --- \| --- \| \| 2 \| training_type_2___2 \| Continuing medical education (CME) ultrasound training course \| \| 3 \| training_type_2___3 \| Seminar during in-person conference \| \| 4 \| training_type_2___4 \| Online course \| \| 5 \| training_type_2___5 \| As part of a study/research project \| \| 6 \| training_type_2___6 \| Other (Please specify) \| |
| 28 | [training_type_other_2]  Show the field ONLY if:  [training_type_2(6)]=1 | Other (Please specify):  *Other type* | text |
| 29 | [training_days_2]  Show the field ONLY if:  [training_number]=2 OR [training_number]=3 OR [training_number]=4 OR [training_number]=5 OR [training_number]=6 OR [training_number]=7 | 9-2c. For your second training, how long was the training? (days)  *days* | text (number) |
| 30 | [training_certification_2]  Show the field ONLY if:  [training_number]=2 OR [training_number]=3 OR [training_number]=4 OR [training_number]=5 OR [training_number]=6 OR [training_number]=7 | 9-2d. For your second training, did you receive some sort of certification? | radio   \| 1 \| Yes \| \| --- \| --- \| \| 2 \| No \| |
| 31 | [training_organization_2]  Show the field ONLY if:  [training_number]=2 OR [training_number]=3 OR [training_number]=4 OR [training_number]=5 OR [training_number]=6 OR [training_number]=7 | 9-2e. For your second training, what organization or who conducted the training?  *Organization* | text |
| 32 | [training_type_3]  Show the field ONLY if:  [training_number]=3 OR [training_number]=4 OR [training_number]=5 OR [training_number]=6 OR [training_number]=7 | 9-3b. For your third training, what type of training did you complete? (Please check all that apply) | checkbox   \| 1 \| training_type_3___1 \| Training occurred during schooling \| \| --- \| --- \| --- \| \| 2 \| training_type_3___2 \| Continuing medical education (CME) ultrasound training course \| \| 3 \| training_type_3___3 \| Seminar during in-person conference \| \| 4 \| training_type_3___4 \| Online course \| \| 5 \| training_type_3___5 \| As part of a study/research project \| \| 6 \| training_type_3___6 \| Other (Please specify) \| |
| 33 | [training_type_other_3]  Show the field ONLY if:  [training_type_3(6)]=1 | Other (Please specify):  *Other type* | text |
| 34 | [training_days_3]  Show the field ONLY if:  [training_number]=3 OR [training_number]=4 OR [training_number]=5 OR [training_number]=6 OR [training_number]=7 | 9-3c. For your third training, how long was the training? (days)  *days* | text (number) |
| 35 | [training_certification_3]  Show the field ONLY if:  [training_number]=3 OR [training_number]=4 OR [training_number]=5 OR [training_number]=6 OR [training_number]=7 | 9-3d. For your third training, did you receive some sort of certification? | radio   \| 1 \| Yes \| \| --- \| --- \| \| 2 \| No \| |
| 36 | [training_organization_3]  Show the field ONLY if:  [training_number]=3 OR [training_number]=4 OR [training_number]=5 OR [training_number]=6 OR [training_number]=7 | 9-3e. For your third training, what organization or who conducted the training?  *Organization* | text |
| 37 | [training_type_4]  Show the field ONLY if:  [training_number]=4 OR [training_number]=5 OR [training_number]=6 OR [training_number]=7 | 9-4b. For your fourth training, what type of training did you complete? (Please check all that apply) | checkbox   \| 1 \| training_type_4___1 \| Training occurred during schooling \| \| --- \| --- \| --- \| \| 2 \| training_type_4___2 \| Continuing medical education (CME) ultrasound training course \| \| 3 \| training_type_4___3 \| Seminar during in-person conference \| \| 4 \| training_type_4___4 \| Online course \| \| 5 \| training_type_4___5 \| As part of a study/research project \| \| 6 \| training_type_4___6 \| Other (Please specify) \| |
| 38 | [training_type_other_4]  Show the field ONLY if:  [training_type_4(6)]=1 | Other (Please specify):  *Other type* | text |
| 39 | [training_days_4]  Show the field ONLY if:  [training_number]=4 OR [training_number]=5 OR [training_number]=6 OR [training_number]=7 | 9-4c. For your fourth training, how long was the training? (days)  *days* | text (number) |
| 40 | [training_certification_4]  Show the field ONLY if:  [training_number]=4 OR [training_number]=5 OR [training_number]=6 OR [training_number]=7 | 9-4d. For your fourth training, did you receive some sort of certification? | radio   \| 1 \| Yes \| \| --- \| --- \| \| 2 \| No \| |
| 41 | [training_organization_4]  Show the field ONLY if:  [training_number]=4 OR [training_number]=5 OR [training_number]=6 OR [training_number]=7 | 9-4e. For your fourth training, what organization or who conducted the training?  *Organization* | text |
| 42 | [training_type_5]  Show the field ONLY if:  [training_number]=5 OR [training_number]=6 OR [training_number]=7 | 9-5b. For your fifth training, what type of training did you complete? (Please check all that apply) | checkbox   \| 1 \| training_type_5___1 \| Training occurred during schooling \| \| --- \| --- \| --- \| \| 2 \| training_type_5___2 \| Continuing medical education (CME) ultrasound training course \| \| 3 \| training_type_5___3 \| Seminar during in-person conference \| \| 4 \| training_type_5___4 \| Online course \| \| 5 \| training_type_5___5 \| As part of a study/research project \| \| 6 \| training_type_5___6 \| Other (Please specify) \| |
| 43 | [training_type_other_5]  Show the field ONLY if:  [training_type_5(6)]=1 | Other (Please specify):  *Other type* | text |
| 44 | [training_days_5]  Show the field ONLY if:  [training_number]=5 OR [training_number]=6 OR [training_number]=7 | 9-5c. For your fifth training, how long was the training? (days)  *days* | text (number) |
| 45 | [training_certification_5]  Show the field ONLY if:  [training_number]=5 OR [training_number]=6 OR [training_number]=7 | 9-5d. For your fifth training, did you receive some sort of certification? | radio   \| 1 \| Yes \| \| --- \| --- \| \| 2 \| No \| |
| 46 | [training_organization_5]  Show the field ONLY if:  [training_number]=5 OR [training_number]=6 OR [training_number]=7 | 9-5e. For your fifth training, what organization or who conducted the training?  *Organization* | text |
| 47 | [training_type_6]  Show the field ONLY if:  [training_number]=6 OR [training_number]=7 | 9-6b. For your sixth training, what type of training did you complete? (Please check all that apply) | checkbox   \| 1 \| training_type_6___1 \| Training occurred during schooling \| \| --- \| --- \| --- \| \| 2 \| training_type_6___2 \| Continuing medical education (CME) ultrasound training course \| \| 3 \| training_type_6___3 \| Seminar during in-person conference \| \| 4 \| training_type_6___4 \| Online course \| \| 5 \| training_type_6___5 \| As part of a study/research project \| \| 6 \| training_type_6___6 \| Other (Please specify) \| |
| 48 | [training_type_other_6]  Show the field ONLY if:  [training_type_6(6)]=1 | Other (Please specify):  *Other type* | text |
| 49 | [training_days_6]  Show the field ONLY if:  [training_number]=6 OR [training_number]=7 | 9-6c. For your sixth training, how long was the training? (days)  *days* | text (number) |
| 50 | [training_certification_6]  Show the field ONLY if:  [training_number]=6 OR [training_number]=7 | 9-6d. For your sixth training, did you receive some sort of certification? | radio   \| 1 \| Yes \| \| --- \| --- \| \| 2 \| No \| |
| 51 | [training_organization_6]  Show the field ONLY if:  [training_number]=6 OR [training_number]=7 | 9-6e. For your sixth training, what organization or who conducted the training?  *Organization* | text |
| 52 | [training_7]  Show the field ONLY if:  [training_number]=7 | 9-7. Please describe any subsequent trainings after your sixth training. | notes |
| 53 | [training_mostrecent]  Show the field ONLY if:  [ultrasoundtraining]=1 | f. How long ago was your most recent training completed? | radio   \| 1 \| Less than 1 month ago \| \| --- \| --- \| \| 2 \| More than 1 month but less than 6 months ago \| \| 3 \| More than 6 months but less than 1 year ago \| \| 4 \| More than 1 year but less than 5 years ago \| \| 5 \| More than 5 years ago \| |
| 54 | [training_confidence]  Show the field ONLY if:  [ultrasoundtraining]=1 | g. After completion of your most recent training, how confident were you in your ability to use ultrasound in clinical care? | radio   \| 1 \| Not confident \| \| --- \| --- \| \| 2 \| Somewhat confident \| \| 3 \| Not sure \| \| 4 \| Confident \| \| 5 \| Very confident \| |
| 55 | [training_6months_confidence]  Show the field ONLY if:  [training_mostrecent]=3 OR [training_mostrecent]=4 OR [training_mostrecent]=5 | h. If your most recent training was completed more than 6 months ago, how confident are you currently in your ability to use ultrasound in clinical care? | radio   \| 1 \| Not confident \| \| --- \| --- \| \| 2 \| Somewhat confident \| \| 3 \| Not sure \| \| 4 \| Confident \| \| 5 \| Very confident \| \| 6 \| Not applicable \| |
| 56 | [training_selftaught]  Show the field ONLY if:  [ultrasoundtraining]=2 | i. If no, are you self-taught on the use of ultrasound? | radio   \| 1 \| Yes \| \| --- \| --- \| \| 2 \| No \| |
| 57 | [selftaught_resources]  Show the field ONLY if:  [training_selftaught]=1 | What resources did you use to teach yourself ultrasound?  *Resources* | notes |
| 58 | [extra_comments] | Please feel free to provide any additional comments or explanations to any of the above questions and/or responses. | notes |
| 59 | [expertise] | Section Header: *Ultrasound Use (22% Completed)*          10. How would you rate your ultrasound expertise? | radio   \| 1 \| No experience \| \| --- \| --- \| \| 2 \| Inexperienced/Novice \| \| 3 \| Somewhat inexperienced \| \| 4 \| Somewhat experienced \| \| 5 \| Very experienced/Expert \| |
| 60 | [trainothers] | 11. Have you been involved in training others on the use of ultrasound? | radio   \| 1 \| Yes \| \| --- \| --- \| \| 2 \| No \| |
| 61 | [currentsettingaccess] | 12. In your current clinical setting, do you have access to ultrasound? | radio   \| 1 \| Yes \| \| --- \| --- \| \| 2 \| No \| |
| 62 | [numberofmachines]  Show the field ONLY if:  [currentsettingaccess]=1 | a. If yes, to how many ultrasound machines?  *Number of machines* | text (integer) |
| 63 | [machinetypes]  Show the field ONLY if:  [currentsettingaccess]=1 | b. If yes, what type(s) ultrasound machines? | radio   \| 1 \| Portable \| \| --- \| --- \| \| 2 \| Not portable \| \| 3 \| Both portable and not portable \| |
| 64 | [portable_handheld]  Show the field ONLY if:  [machinetypes]=1 OR [machinetypes]=3 | Portable: Is this handheld? | radio   \| 1 \| Yes \| \| --- \| --- \| \| 2 \| No \| |
| 65 | [probes]  Show the field ONLY if:  [currentsettingaccess]=1 | c. If yes, what type of ultrasound probes do you have? (Please check all that apply) | checkbox   \| 1 \| probes___1 \| Linear \| \| --- \| --- \| --- \| \| 2 \| probes___2 \| Curved \| \| 3 \| probes___3 \| Phased Array \| \| 4 \| probes___4 \| Endocavitary \| \| 5 \| probes___5 \| Multi-use probe \| \| 6 \| probes___6 \| Other (Please specify) \| |
| 66 | [probes_other]  Show the field ONLY if:  [probes(6)]=1 | Other (Please specify):  *Other ultrasound probe* | notes |
| 67 | [ultrasounduse] | 13. How often do you use ultrasound in your clinical practice? | radio   \| 1 \| Daily \| \| --- \| --- \| \| 2 \| Weekly \| \| 3 \| Monthly \| \| 4 \| Every few months \| \| 5 \| Rarely \| \| 6 \| Never \| |
| 68 | [ultrasounduse_setting] | 14. What do you use ultrasound for in your clinical setting? (Please check all that apply) | checkbox   \| 1 \| ultrasounduse_setting___1 \| Obstetrics/gynecology \| \| --- \| --- \| --- \| \| 2 \| ultrasounduse_setting___2 \| Trauma/FAST \| \| 3 \| ultrasounduse_setting___3 \| Cardiac/echocardiography \| \| 4 \| ultrasounduse_setting___4 \| Abdominal aortic aneurysm \| \| 5 \| ultrasounduse_setting___5 \| Gallbladder \| \| 6 \| ultrasounduse_setting___6 \| Renal/Bladder \| \| 7 \| ultrasounduse_setting___7 \| Lung \| \| 8 \| ultrasounduse_setting___8 \| Musculoskeletal \| \| 9 \| ultrasounduse_setting___9 \| Skin and soft tissue \| \| 10 \| ultrasounduse_setting___10 \| Nerve blocks \| \| 11 \| ultrasounduse_setting___11 \| Central line insertion \| \| 12 \| ultrasounduse_setting___12 \| Thoracentesis \| \| 13 \| ultrasounduse_setting___13 \| Paracentesis \| \| 14 \| ultrasounduse_setting___14 \| Pericardiocentesis \| \| 15 \| ultrasounduse_setting___15 \| Other (Please specify) \| |
| 69 | [ultrasounduse_setting_other_specify]  Show the field ONLY if:  [ultrasounduse_setting(15)]=1 | Other (Please specify):  *Other ultrasound use* | text |
| 70 | [extra_comments_ultrasound_use] | Please feel free to provide any additional comments or explanations to any of the above questions and/or responses. | notes |
| 71 | [access_machine] | Section Header: *Barriers/Facilitators (34% Completed) Please note whether you consider the following factors barriers (prevents you from using ultrasound) or facilitators (enables you using ultrasound) to the use of ultrasound in the care of your patients.*  15. Access to an ultrasound machine | radio (Matrix)   \| 1 \| Definitely a barrier \| \| --- \| --- \| \| 2 \| Somewhat a barrier \| \| 3 \| Neither a barrier nor facilitator \| \| 4 \| Somewhat a facilitator \| \| 5 \| Definitely a facilitator \| \| 6 \| Not applicable \| |
| 72 | [access_probe] | 16. Access to an ultrasound probe | radio (Matrix)   \| 1 \| Definitely a barrier \| \| --- \| --- \| \| 2 \| Somewhat a barrier \| \| 3 \| Neither a barrier nor facilitator \| \| 4 \| Somewhat a facilitator \| \| 5 \| Definitely a facilitator \| \| 6 \| Not applicable \| |
| 73 | [access_maintenance] | 17. Access to maintenance/repair for ultrasound machines and/or probes | radio (Matrix)   \| 1 \| Definitely a barrier \| \| --- \| --- \| \| 2 \| Somewhat a barrier \| \| 3 \| Neither a barrier nor facilitator \| \| 4 \| Somewhat a facilitator \| \| 5 \| Definitely a facilitator \| \| 6 \| Not applicable \| |
| 74 | [access_gel] | 18. Access to ultrasound gel | radio (Matrix)   \| 1 \| Definitely a barrier \| \| --- \| --- \| \| 2 \| Somewhat a barrier \| \| 3 \| Neither a barrier nor facilitator \| \| 4 \| Somewhat a facilitator \| \| 5 \| Definitely a facilitator \| \| 6 \| Not applicable \| |
| 75 | [access_electricity] | 19. Access to reliable electricity/power | radio (Matrix)   \| 1 \| Definitely a barrier \| \| --- \| --- \| \| 2 \| Somewhat a barrier \| \| 3 \| Neither a barrier nor facilitator \| \| 4 \| Somewhat a facilitator \| \| 5 \| Definitely a facilitator \| \| 6 \| Not applicable \| |
| 76 | [access_education] | 20. Access to ultrasound education and/or training | radio (Matrix)   \| 1 \| Definitely a barrier \| \| --- \| --- \| \| 2 \| Somewhat a barrier \| \| 3 \| Neither a barrier nor facilitator \| \| 4 \| Somewhat a facilitator \| \| 5 \| Definitely a facilitator \| \| 6 \| Not applicable \| |
| 77 | [access_expertsupport] | 21. Access to support from an ultrasound expert for ongoing mentoring and training | radio (Matrix)   \| 1 \| Definitely a barrier \| \| --- \| --- \| \| 2 \| Somewhat a barrier \| \| 3 \| Neither a barrier nor facilitator \| \| 4 \| Somewhat a facilitator \| \| 5 \| Definitely a facilitator \| \| 6 \| Not applicable \| |
| 78 | [access_resources] | 22. Access to ultrasound learning resources | radio (Matrix)   \| 1 \| Definitely a barrier \| \| --- \| --- \| \| 2 \| Somewhat a barrier \| \| 3 \| Neither a barrier nor facilitator \| \| 4 \| Somewhat a facilitator \| \| 5 \| Definitely a facilitator \| \| 6 \| Not applicable \| |
| 79 | [access_consultants] | 23. Access to radiology consultants for ultrasound image acquisition and/or interpretation | radio (Matrix)   \| 1 \| Definitely a barrier \| \| --- \| --- \| \| 2 \| Somewhat a barrier \| \| 3 \| Neither a barrier nor facilitator \| \| 4 \| Somewhat a facilitator \| \| 5 \| Definitely a facilitator \| \| 6 \| Not applicable \| |
| 80 | [policies] | 24. Policies and/or guidelines on ultrasound use | radio (Matrix)   \| 1 \| Definitely a barrier \| \| --- \| --- \| \| 2 \| Somewhat a barrier \| \| 3 \| Neither a barrier nor facilitator \| \| 4 \| Somewhat a facilitator \| \| 5 \| Definitely a facilitator \| \| 6 \| Not applicable \| |
| 81 | [competition] | 25. Competition for ultrasound use with other providers and/or departments | radio (Matrix)   \| 1 \| Definitely a barrier \| \| --- \| --- \| \| 2 \| Somewhat a barrier \| \| 3 \| Neither a barrier nor facilitator \| \| 4 \| Somewhat a facilitator \| \| 5 \| Definitely a facilitator \| \| 6 \| Not applicable \| |
| 82 | [acceptance] | 26. Staff/other providers' acceptance of ultrasound as an appropriate imaging tool | radio (Matrix)   \| 1 \| Definitely a barrier \| \| --- \| --- \| \| 2 \| Somewhat a barrier \| \| 3 \| Neither a barrier nor facilitator \| \| 4 \| Somewhat a facilitator \| \| 5 \| Definitely a facilitator \| \| 6 \| Not applicable \| |
| 83 | [support] | 27. Hospital administration's support of ultrasound as an appropriate imaging tool | radio (Matrix)   \| 1 \| Definitely a barrier \| \| --- \| --- \| \| 2 \| Somewhat a barrier \| \| 3 \| Neither a barrier nor facilitator \| \| 4 \| Somewhat a facilitator \| \| 5 \| Definitely a facilitator \| \| 6 \| Not applicable \| |
| 84 | [sendimages] | 28. Ability to send ultrasound images for interpretation by a remote ultrasound expert | radio (Matrix)   \| 1 \| Definitely a barrier \| \| --- \| --- \| \| 2 \| Somewhat a barrier \| \| 3 \| Neither a barrier nor facilitator \| \| 4 \| Somewhat a facilitator \| \| 5 \| Definitely a facilitator \| \| 6 \| Not applicable \| |
| 85 | [generateimages] | 29. Your ability to generate high-quality ultrasound images | radio (Matrix)   \| 1 \| Definitely a barrier \| \| --- \| --- \| \| 2 \| Somewhat a barrier \| \| 3 \| Neither a barrier nor facilitator \| \| 4 \| Somewhat a facilitator \| \| 5 \| Definitely a facilitator \| \| 6 \| Not applicable \| |
| 86 | [interpretimages] | 30. Your ability to interpret ultrasound images | radio (Matrix)   \| 1 \| Definitely a barrier \| \| --- \| --- \| \| 2 \| Somewhat a barrier \| \| 3 \| Neither a barrier nor facilitator \| \| 4 \| Somewhat a facilitator \| \| 5 \| Definitely a facilitator \| \| 6 \| Not applicable \| |
| 87 | [examtime] | 31. Amount of time it takes to conduct an ultrasound exam | radio (Matrix)   \| 1 \| Definitely a barrier \| \| --- \| --- \| \| 2 \| Somewhat a barrier \| \| 3 \| Neither a barrier nor facilitator \| \| 4 \| Somewhat a facilitator \| \| 5 \| Definitely a facilitator \| \| 6 \| Not applicable \| |
| 88 | [referability] | 32. Ability to refer patients to higher level of care for management of ultrasound findings | radio (Matrix)   \| 1 \| Definitely a barrier \| \| --- \| --- \| \| 2 \| Somewhat a barrier \| \| 3 \| Neither a barrier nor facilitator \| \| 4 \| Somewhat a facilitator \| \| 5 \| Definitely a facilitator \| \| 6 \| Not applicable \| |
| 89 | [patientunderstanding] | 33. Patient's understanding of ultrasound technology | radio (Matrix)   \| 1 \| Definitely a barrier \| \| --- \| --- \| \| 2 \| Somewhat a barrier \| \| 3 \| Neither a barrier nor facilitator \| \| 4 \| Somewhat a facilitator \| \| 5 \| Definitely a facilitator \| \| 6 \| Not applicable \| |
| 90 | [patientacceptance] | 34. Patient's acceptance of having ultrasound performed on them | radio (Matrix)   \| 1 \| Definitely a barrier \| \| --- \| --- \| \| 2 \| Somewhat a barrier \| \| 3 \| Neither a barrier nor facilitator \| \| 4 \| Somewhat a facilitator \| \| 5 \| Definitely a facilitator \| \| 6 \| Not applicable \| |
| 91 | [cost] | 35. Cost of ultrasound machine/probe/equipment | radio (Matrix)   \| 1 \| Definitely a barrier \| \| --- \| --- \| \| 2 \| Somewhat a barrier \| \| 3 \| Neither a barrier nor facilitator \| \| 4 \| Somewhat a facilitator \| \| 5 \| Definitely a facilitator \| \| 6 \| Not applicable \| |
| 92 | [machineprobe_barrier]  Show the field ONLY if:  [access_machine]=1 or [access_machine]=2 or [access_probe]=1 or [access_probe]=2 | If access to an ultrasound machine or probe is somewhat or definitely a barrier, why is it a barrier? (Please check all that apply) | checkbox   \| 1 \| machineprobe_barrier___1 \| Competition for use by individuals in same department \| \| --- \| --- \| --- \| \| 2 \| machineprobe_barrier___2 \| Competition for use by individuals in different departments \| \| 3 \| machineprobe_barrier___3 \| Ultrasound machine is not available \| \| 4 \| machineprobe_barrier___4 \| Ultrasound probe is not available \| \| 5 \| machineprobe_barrier___5 \| Other (Please specify) \| |
| 93 | [machineprobe_barrier_other]  Show the field ONLY if:  [machineprobe_barrier(5)]=1 | Other (Please specify):  *Other reason* | text |
| 94 | [expertsupport_location]  Show the field ONLY if:  [access_expertsupport]=4 or [access_expertsupport]=5 | If access to support from an ultrasound expert for ongoing mentoring and training is somewhat or definitely an enabler, is the ultrasound expert local or remote? | radio   \| 1 \| Local \| \| --- \| --- \| \| 2 \| Remote \| |
| 95 | [biggestbarrier] | 36. What is the biggest barrier (if any) to ultrasound use in your setting? | radio   \| 1 \| Access to an ultrasound machine \| \| --- \| --- \| \| 2 \| Access to an ultrasound probe \| \| 3 \| Access to maintenance/repair for ultrasound machines and/or probes \| \| 4 \| Access to ultrasound gel \| \| 5 \| Access to reliable electricity and/or power \| \| 6 \| Access to ultrasound education and/or training \| \| 7 \| Access to support from an ultrasound expert for ongoing mentoring and training \| \| 8 \| Access to ultrasound learning resources \| \| 9 \| Access to radiology consultants for ultrasound image acquisition and/or interpretation \| \| 10 \| Policies and/or guidelines on ultrasound use \| \| 11 \| Competition for ultrasound use with other providers and/or departments \| \| 12 \| Staff/other providers' acceptance of ultrasound as an appropriate imaging tool \| \| 13 \| Hospital administration's support of ultrasound as an appropriate imaging tool \| \| 14 \| Ability to send ultrasound images for interpretation by a remote ultrasound expert \| \| 15 \| Your ability to generate high-quality ultrasound images \| \| 16 \| Your ability to interpret ultrasound images \| \| 17 \| Amount of time it takes to conduct an ultrasound exam \| \| 18 \| Ability to refer patients to higher level of care for management of ultrasound findings \| \| 19 \| Patient's understanding of ultrasound technology \| \| 20 \| Patient's acceptance of having ultrasound performed on them \| \| 21 \| Cost of ultrasound machine/probe/equipment \| \| 22 \| Other (Please specify) \| \| 23 \| Not applicable \| |
| 96 | [biggestbarrier_other]  Show the field ONLY if:  [biggestbarrier]=22 | Other (Please specify)  *Other barrier* | notes |
| 97 | [biggestfacilitator] | 37.  What is the biggest facilitator (if any) to ultrasound use in your setting? | radio   \| 1 \| Access to an ultrasound machine \| \| --- \| --- \| \| 2 \| Access to an ultrasound probe \| \| 3 \| Access to maintenance/repair for ultrasound machines and/or probes \| \| 4 \| Access to ultrasound gel \| \| 5 \| Access to reliable electricity and/or power \| \| 6 \| Access to ultrasound education and/or training \| \| 7 \| Access to support from an ultrasound expert for ongoing mentoring and training \| \| 8 \| Access to ultrasound learning resources \| \| 9 \| Access to radiology consultants for ultrasound image acquisition and/or interpretation \| \| 10 \| Policies and/or guidelines on ultrasound use \| \| 11 \| Competition for ultrasound use with other providers and/or departments \| \| 12 \| Staff/other providers' acceptance of ultrasound as an appropriate imaging tool \| \| 13 \| Hospital administration's support of ultrasound as an appropriate imaging tool \| \| 14 \| Ability to send ultrasound images for interpretation by a remote ultrasound expert \| \| 15 \| Your ability to generate high-quality ultrasound images \| \| 16 \| Your ability to interpret ultrasound images \| \| 17 \| Amount of time it takes to conduct an ultrasound exam \| \| 18 \| Ability to refer patients to higher level of care for management of ultrasound findings \| \| 19 \| Patient's understanding of ultrasound technology \| \| 20 \| Patient's acceptance of having ultrasound performed on them \| \| 21 \| Cost of ultrasound machine/probe/equipment \| \| 22 \| Other (Please specify) \| \| 23 \| Not applicable \| |
| 98 | [biggestfacilitator_other]  Show the field ONLY if:  [biggestfacilitator]=22 | Other (Please specify)  *Other facilitator* | notes |
| 99 | [barrier_enablers_workplace_policies] | 38. Does your location or place of work have policies and/or guidelines for ultrasound use? | radio   \| 1 \| Yes \| \| --- \| --- \| \| 2 \| No \| |
| 100 | [barrier_enablers_comments] | Please feel free to provide any additional comments or explanations to any of the above questions and/or responses. | notes |
| 101 | [accessimportance] | Section Header: *Importance of Ultrasound (93% Completed)*        39. How important is having access to ultrasound for patient care? | radio   \| 1 \| Very unimportant \| \| --- \| --- \| \| 2 \| Unimportant \| \| 3 \| Neither important nor unimportant \| \| 4 \| Important \| \| 5 \| Very important \| |
| 102 | [qualityofcare] | 40. Ultrasound improves the quality of care for patients: | radio   \| 1 \| Strongly disagree \| \| --- \| --- \| \| 2 \| Disagree \| \| 3 \| Neither agree nor disagree \| \| 4 \| Agree \| \| 5 \| Strongly agree \| |
| 103 | [morbidity] | 41. Ultrasound improves patient outcomes: | radio   \| 1 \| Strongly disagree \| \| --- \| --- \| \| 2 \| Disagree \| \| 3 \| Neither agree nor disagree \| \| 4 \| Agree \| \| 5 \| Strongly agree \| |
| 104 | [fears] | 42. Do you have any fears/reservations associated with using ultrasound in a clinical setting? | radio   \| 1 \| Yes \| \| --- \| --- \| \| 2 \| No \| |
| 105 | [fears_elaborate]  Show the field ONLY if:  [fears]=1 | If yes, please elaborate: | checkbox   \| 1 \| fears_elaborate___1 \| Misdiagnosis \| \| --- \| --- \| --- \| \| 2 \| fears_elaborate___2 \| Patients not understanding technology \| \| 3 \| fears_elaborate___3 \| Leadership/administration not supporting use \| \| 4 \| fears_elaborate___4 \| Peers not supporting use \| \| 5 \| fears_elaborate___5 \| Malpractice \| \| 6 \| fears_elaborate___6 \| Other (Please specify) \| |
| 106 | [fears_elaborate_other]  Show the field ONLY if:  [fears_elaborate(6)]=1 | Other (Please specify):  *Other fears/reservations* | notes |
| 107 | [extra_comments_final] | Please feel free to provide any additional comments or explanations to any of the above questions and/or responses. | notes |
| 108 | [ultrasound_barriers_facilitators_complete] | Section Header: *Form Status*  Complete? | dropdown   \| 0 \| Incomplete \| \| --- \| --- \| \| 1 \| Unverified \| \| 2 \| Complete \| |
